# Hemizygous Granzyme A Mice Expressing the hSOD1G93A Transgene Show Slightly Extended Lifespan

**DOI:** 10.3390/ijms232113554

**Published:** 2022-11-04

**Authors:** Laura Moreno-Martinez, Llipsy Santiago, Miriam de la Torre, Ana Cristina Calvo, Julián Pardo, Rosario Osta

**Affiliations:** 1 LAGENBIO, Faculty of Veterinary, University of Zaragoza, Miguel Servet 177, 50013 Zaragoza, Spain; 2 Centre for Biomedical Research in Neurodegenerative Diseases (CIBERNED), Instituto de Salud Carlos III, 28029 Madrid, Spain; 3 Biomedical Research Centre of Aragón (CIBA), Aragón Health Research Institute (IIS Aragón), 50009 Zaragoza, Spain; 4 AgriFood Institute of Aragon-IA2 (UNIZAR-CITA), 50013 Zaragoza, Spain; 5 Centro de Investigación Biomédica en Red de Enfermedades Infecciosas (CIBERINFEC), Instituto de Salud Carlos III, 28029 Madrid, Spain; 6 Department of Microbiology, Preventive Medicine and Public Health, University of Zaragoza, 50009 Zaragoza, Spain

**Keywords:** granzyme A, SOD1G93A mouse, inflammation, glutathione reductase, amyotrophic lateral sclerosis

## Abstract

Granzyme A (gzmA), a serine protease involved in the modulation of the inflammatory immune response, is found at an elevated level in the serum from ALS patients. However, the influence of gzmA on the progression of ALS remains unclear. The aim of our work was to assess whether the absence of gzmA in an ALS murine model could help slow down the progression of the disease. Homozygous and hemizygous gzmA-deficient mice expressing the hSOD1G93A transgene were generated, and survival of these mice was monitored. Subsequently, gene and protein expression of inflammatory and oxidative stress markers was measured in the spinal cord and quadriceps of these mice. We observed the longest lifespan in gzmA+/− mice. GzmA gene and protein expression was downregulated in the spinal cord and serum from gmzA+/− mice, confirming that the increased survival of hemizygous mice is correlated with lower levels of gzmA. In addition, mRNA and protein levels of glutathione reductase (GSR), involved in oxidative stress, were found downregulated in the spinal cord and quadriceps of gmzA+/− mice, together with lower IL-1β and IL-6 mRNA levels in hemyzigous mice. In summary, our findings indicate for the first time that reduced levels, but not the absence, of gzmA could slightly ameliorate the disease progression in this animal model.

## 1. Introduction

Amyotrophic lateral sclerosis (ALS) is a devastating neurodegenerative disease that leads to progressive motor neuron degeneration and muscle denervation [1]. Only 5–10% of ALS patients have a family history, whereas more than 90% of ALS cases are sporadic with an unknown cause. 

One of the emerging mechanisms involved in ALS pathogenesis is neuroinflammation [2,3,4,5,6]. This mechanism is characterized by a progressive transition from microglia type M2 to M1, where infiltrating peripheral monocytes and lymphocytes are found together with the release of inflammatory cytokines and chemokines, resulting in inflammation and neuronal death [7,8,9]. This pathway has been extensively studied in animal models of ALS, especially in the high-copy transgenic SOD1G93A mouse model, which overexpresses the mutated human superoxide dismutase 1 enzyme, with a change of glycine to alanine at codon 93 (SOD1G93A). Currently, this animal model is considered one of the best murine models of ALS, as it develops phenotypic features similar to those seen in ALS patients [10]. In the SOD1G93A mouse model, both pro-inflammatory and anti-inflammatory cytokines, such as tumor necrosis factor alpha (TNF-α), interleukin (IL)-1β, IL-2, IL-6 and IL-10, have been found at elevated levels in blood, especially at the final stage, pointing out an evident failure to counteract inflammation in this model [11,12,13]. Inflammation has also been studied in other tissues that are damaged in ALS. For instance, microglial inflammatory markers CD11b and CD68, and inflammatory cytokines IL-1β and TNF-α were found increased in the skeletal muscle of symptomatic and end-stage SOD1G93A rats [14]. Similarly, elevated levels of transforming growth factor beta (TGF-β) were found in skeletal muscles of ALS patients and the SOD1G93A mouse model and were correlated with disease progression, suggesting this cytokine is a muscle biomarker for disease progression in ALS [15].

Apart from cytokines, immune cells such as T cells also play an important role in the pathogenesis of ALS. For instance, CD4+ T helper and CD8+ T suppressor/cytotoxic cells were found at elevated levels in the post-mortem spinal cord of ALS patients, supporting the impaired regulation of the immune system in this tissue in ALS [16,17]. Cytotoxic CD8+ T cells are capable of attacking target cells via the release of cytotoxic proteins such as perforin and granule-secreted enzymes (granzymes) [18]. Granzymes belong to the family of serine proteases which are mainly expressed by T lymphocytes and natural killer (NK) cells [19,20]. Traditionally, they were thought to exert their biological functions by inducing cell death in tumor and virus-infected cells. However, in the last years, it has been shown that they can also regulate other biological processes, including the modulation of the inflammatory immune response during different inflammatory/autoimmune diseases such as sepsis, aging, cardiovascular diseases or arthritis [19,20,21,22]. Specifically, it has been demonstrated that granzyme A (gzmA) can induce a pro-inflammatory cytokine response after stimulating monocytic cells, causing the secretion of IL-1β, IL-6 and TNF-α [23,24]. Furthermore, inhibition of gzmA with serpinb6b, a specific inhibitor of mouse gzmA, can reduce inflammation by regulating the expression of IL-6 [25], highlighting gzmA as a key regulator of the immune response. Supporting the involvement of granzymes in ALS, one study reported that activated interferon gamma (IFNγ)-producing SOD1G93A CD8+ T cells can induce the death of motoneurons through granzyme pathways [26], suggesting that granzymes could play a harmful role in ALS. Accordingly, both gzmA and granzyme B (gzmB) serum levels were found elevated in ALS patients and were associated with faster progression of the disease [27].

Here we hypothesized that, as gzmA is responsible for the secretion of a pool of pro-inflammatory cytokines, modulation of this molecule could allow us to indirectly target multiple cytokines that have been demonstrated to be damaging in ALS. Considering this, the objective of this work was to assess whether the absence of gzmA could help slow down the course of ALS in vivo. In this study, we demonstrated that a partial deficiency of gzmA may slightly ameliorate the progression of the disease.

## 2. Results

### 2.1. Lifespan Is Extended in Hemizygous gzmA+/− Mice

**Figure 1:**
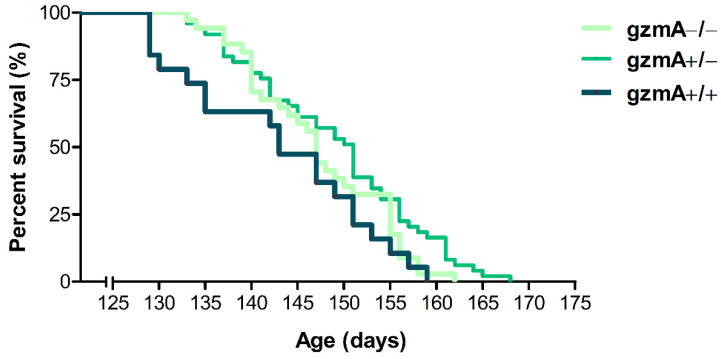
Survival analysis of SOD1G93A mice varying in gzmA genotype. Survival analysis was performed with mice showing the three gzmA genotypes *(n* = 34 gzmA−/−, *n* = 49 gzmA+/− and *n* = 19 gzmA+/+). Survival curve showing statistically significant differences in the lifespan between gzmA+/− and gzmA+/+ mice (*p* = 0.022).

Our aim was to assess whether deficiency of gzmA could have an effect on disease progression and lifespan of SOD1G93A transgenic mice. First, hemizygous SOD1G93A males (SOD1+/−, gzmA+/+) were crossed with C57BL/6 females deficient in gzmA (SOD1−/−, gzmA−/−) to obtain littermate mice with the three gzmA genotypes (gzmA+/+, gzmA+/− and gzmA−/−) in the same genetic background in F2. Then, we analyzed the lifespan of these three groups of mice expressing the hSOD1G93A transgene. Descriptive statistics of the survival analysis for each group are shown in Table 1. As a result, gzmA+/− mice lived slightly longer than the gzmA+/+ group (*p* = 0.022). In contrast, no statistically significant differences were found between survival rates of either gzmA−/− mice and gzmA+/+ (*p* = 0.096) or gzmA−/− and gzmA+/− (*p* = 0.339) (Figure 1).

### 2.2. Human Mutant Superoxide Dismutase 1 (hSOD1G93A) Is Consistently Expressed in All Mice, Regardless of gzmA Genotype

**Figure 2:**
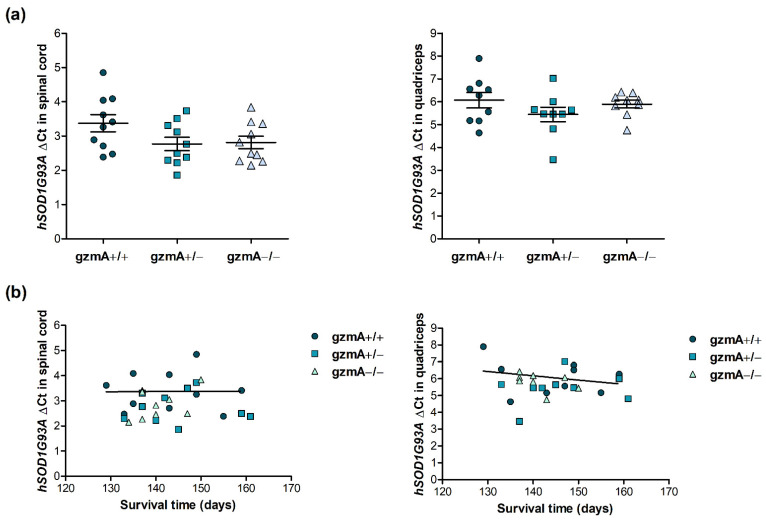
*hSOD1G93A* expression in spinal cord and quadriceps of gzmA+/+, gzmA+/− and gzmA−/− mice involved in the survival assay. (**a**) Expression of *hSOD1G93A* was quantified in both tissues from 10 gmzA+/+, 10 gzmA+/− and 10 gzmA−/− mice. No differences between genotypes were found (*p* > 0.05). (**b**) Correlations between *hSOD1G93A* expression and survival time were not statistically significant (r^2^ = 0.0000884, *p* = 0.979 in spinal cord, r^2^ = 0.0649, *p* = 0.508 in quadriceps).

After the survival analysis, we analyzed hSOD1G93A gene expression to confirm that the mutant gene was consistently expressed in all mice including the three gzmA genotypes and it did not have an effect on the lifespan of mice. hSOD1G93A expression was quantified by reverse transcription quantitative real-time PCR (RT-qPCR) in the spinal cord and quadricep muscle of 10 gmzA+/+, 10 gzmA+/− and 10 gzmA−/− mice from the survival assay. We could not observe any difference in hSOD1G93A expression in any of the tissues when we compared the levels obtained in the three gmzA genotypes (*p* > 0.05) (Figure 2a). Accordingly, no correlation was found between hSOD1G93A expression and the survival time of mice (r^2^ = 0.0000884, *p* = 0.979 in spinal cord, r^2^ = 0.0649, *p* = 0.508 in quadriceps) (Figure 2b).

### 2.3. GzmA Is Expressed According to Each gzmA Genotype

**Figure 3:**
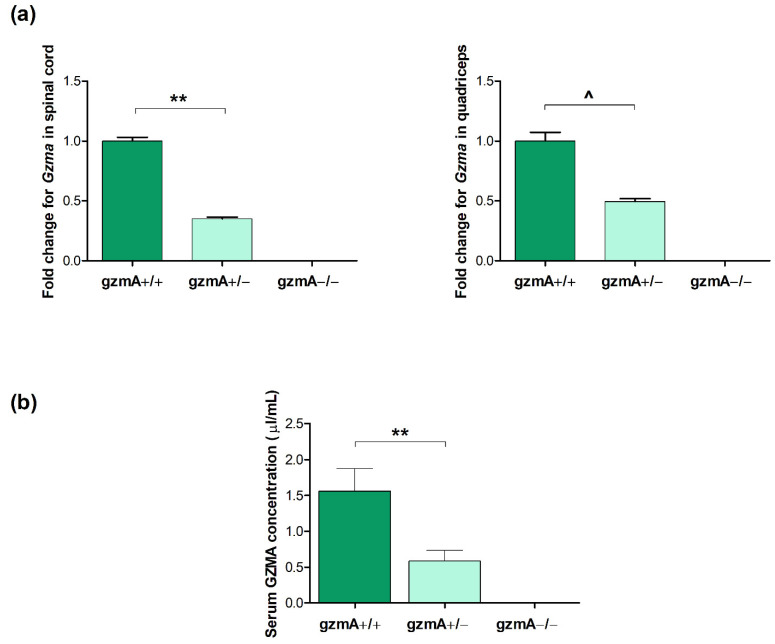
GzmA gene and protein expression in serum, spinal cord and quadriceps from SOD1G93A mice. (**a**) Gzma gene expression was analyzed in spinal cord and quadriceps of 16 SOD1+/− mice from the survival assay, including gzmA+/+(*n* = 6), gzmA+/− (*n* = 6) and gzmA−/− (*n* = 4) mice. Gzma levels were found downregulated in spinal cord from gzmA+/− mice compared to the levels found in gzmA+/+ mice (*p* = 0.001). Similar tendency was observed in quadricep tissue (*p* = 0.076). (**b**) Serum gzmA was measured in 33 SOD1+/− mice at 90 days old, including gzmA+/+ (*n* = 7), gzmA+/− (*n* = 14) and gzmA−/− (*n* = 12) genotypes. Serum gzmA levels were found reduced in gzmA+/− mice (*p* = 0.005). Bars represent mean ± standard error of the mean. ** *p* < 0.01, ^ *p* < 0.10.

Considering the different survival time of mice depending on the gzmA genotype, we aimed to confirm that those variations were due to a real downregulation of gzmA gene and protein expression. In this sense, we analyzed gzmA levels in the serum, spinal cord and quadricep muscle. The RT-qPCR assay showed that gzmA messenger RNA (mRNA) levels were statistically significantly reduced in the spinal cord of gzmA+/− mice compared to the levels obtained from gzmA+/+ mice (*p* = 0.001). As expected, no *Gzma* expression was detected in the gzmA knockout mice used as controls (Figure 3a). In quadriceps, we could observe a similar tendency in hemizygous mice (*p* = 0.076) (Figure 3a). Similarly, serum gzmA levels were found higher in gzmA+/+ mice than in hemizygous mice (*p* = 0.005) (Figure 3b), whereas no gzmA protein was quantified in gmzA−/− mice (Figure 3b).

### 2.4. Gene Expression of Il1b, Il6, Tnfa and Glutathione Reductase (Gsr) Is Reduced in Spinal Cord or Quadricep Muscle of Hemizygous gzmA Mice

**Figure 4:**
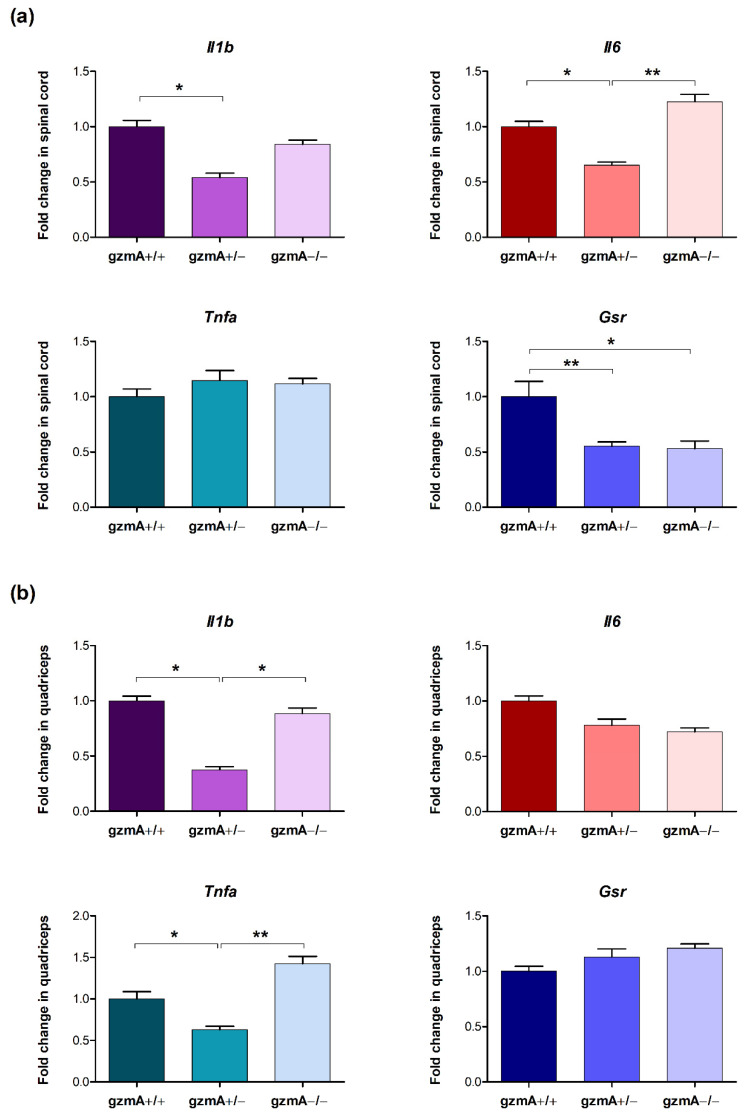
*Il1b*, *Il6*, *Tnfa* and *Gsr* gene expression in spinal cord (**a**) and quadriceps (**b**) from SOD1G93A mice. *Il1b*, *Il6, Tnfa* and *Gsr* gene expression was analyzed in spinal cord and quadriceps of 30 SOD1+/− mice, including gzmA+/+ (*n* = 10), gzmA+/− (*n* = 10) and gzmA−/− (*n* = 10) mice from the survival assay. (**a**) *Il1b*, *Il6* and *Gsr* levels were found reduced in spinal cord from gzmA+/− mice. (**b**) *Il1b* and *Tnfa* levels were reduced in quadriceps from gzmA+/− mice. Bars represent mean ± standard error of the mean. ** *p* < 0.01, * *p* < 0.05.

Next, we studied how reduced levels of gzmA could have an effect on gzmA-regulated genes involved in inflammation. In this sense, we analyzed mRNA expression of *Il1b, Il6* and *Tnfa* in spinal cord and quadricep muscle from mice involved in the survival study. The RT-qPCR assay showed that both *Il1b* and *Il6* levels were statistically significantly lower in the spinal cord of gzmA+/− mice (*p* < 0.05) (Figure 4a). In quadricep tissue, *Il1b* and *Tnfa* expression was reduced in hemizygous mice (*p* < 0.05) (Figure 4b).

To study the effect of the reduction of gmzA in more depth, we measured mRNA levels of *Gsr*, involved in oxidative stress, which is a relevant mechanism dysregulated in ALS disease. Our results show a downregulation of *Gsr* levels in the spinal cord of gzmA+/− and gzmA−/− mice compared to the levels obtained in homozygous gzmA+/+ mice (*p* < 0.05) (Figure 4a). In contrast, similar levels of *Gsr* were obtained in quadriceps in all the mice (Figure 4b). 

### 2.5. Protein Levels of GSR Are Reduced in Spinal Cord and Quadriceps of Hemizygous gzmA Mice

**Figure 5:**
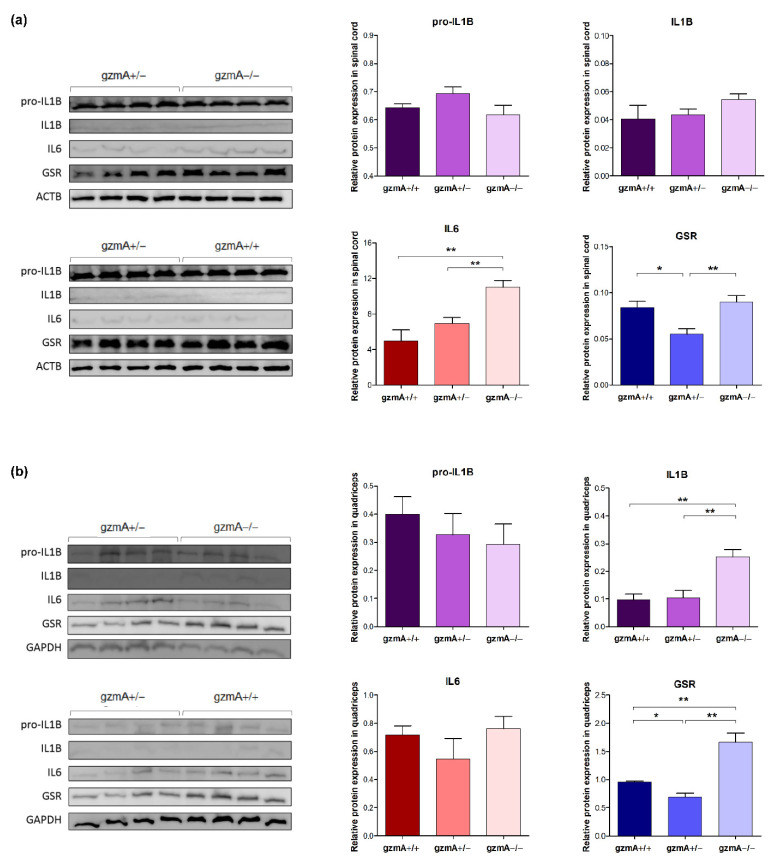
IL1B, IL6 and GSR protein expression in spinal cord (**a**) and quadriceps (**b**) from SOD1G93A mice from the survival assay. IL1Β, IL6 and GSR protein expression was analyzed in spinal cord and quadriceps of 12 SOD1+/− mice, including gzmA+/+(*n* = 4), gzmA+/− (*n* = 4) and gzmA−/− (*n* = 4) mice. GSR was found at lower levels in hemizygous mice compared to both homozygous mice. Levels of IL6 in spinal cord and IL1B in quadriceps were reduced in gzmA+/+ and gzmA+/− mice. ** *p* < 0.01, * *p* < 0.05.

After studying mRNA levels of *Il1b*, *Il6* and *Gsr*, we also analyzed their expression at protein level in the same tissues by western blot. Similar to the results obtained in mRNA, GSR was significantly downregulated in hemizygous mice in both the spinal cord and quadriceps (Figure 5). However, the pro-inflammatory cytokines IL6 and IL1B were reduced only in the spinal cord and quadriceps, respectively, of both gzmA +/+ and gzmA +/− mice (Figure 5). Protein expression of TNFA was measured by ELISA, but no expression could be quantified in any of the studied tissues.

## 3. Discussion

Neuroinflammation is one of the hallmarks in ALS, in which an inflammatory state prevails as the disease progresses. Among the very well-known granzymes in humans, gzmA mainly potentiates the release of cytokines to the extracellular environment, promoting a pro-inflammatory response [24,28]. GzmA can also participate in inflammatory and autoimmune diseases [22,29,30]. In this line, reduced levels of pro-inflammatory cytokines were found in gzmA knockout mouse models of rheumatoid arthritis and sepsis [31,32]. Regarding ALS, elevated serum gzmA levels have been found in ALS patients, suggesting that the fine-tuned modulation of gzmA response may counteract inflammation in ALS [27]. In this work, we found that reduced levels, but not the total absence, of gzmA was sufficient to slightly slow down the course of the disease and regulate inflammatory cytokines and an enzyme, GSR, involved in the oxidative stress mechanism.

First, we crossed hemizygous SOD1G93A males (SOD1+/−, gzmA+/+) with C57BL/6 females deficient in gzmA (SOD1−/−, gzmA−/−) to generate mice showing the three gzmA genotypes (gzmA+/+, gzmA+/− and gzmA−/−) in the same genetic background. Then, these mice were monitored until their humane endpoint, and their survival time was recorded. Contrary to expectations, hemizygous mice, and not those mice deficient in gzmA, showed the greatest survival rate. This result may suggest that a partial downregulation of gzmA could be sufficient to slightly ameliorate the course of the disease. Therefore, gzmA could be partially beneficial for the disease, despite its active role in inflammation processes.

Following the survival analysis, *hSOD1G93A* gene expression was analyzed in the new transgenic mouse to evaluate whether the transgene causing the ALS phenotype was consistently expressed in all the mice, regardless of their gzmA genotype. Previous studies have shown that low-copy mouse models carrying from eight to ten copies of the transgene showed a slower course of disease [33,34]. In this study, *hSOD1G93A* mRNA was found at similar levels in both the spinal cord and quadriceps, and no correlation was found between *hSOD1G93A* mRNA levels and the lifespan either, confirming the consistent *hSOD1G93A* expression in all mice.

Expression of gzmA was also measured to evaluate whether its levels were associated and correlated to each gzmA genotype. Our results show lower mRNA and protein levels of gzmA in the spinal cord and serum from hemizygous mice compared to gzmA+/+ mice. As expected, gzmA was not expressed in mice deficient in gzmA. This result confirms that reduced expression of gzmA is in accordance with the observed extended lifespan of gzmA+/− mice. To support our results and assess the biological effect of gmzA reduction in this mouse model, gzmA-related cytokines were analyzed, particularly IL-1β, IL-6 and TNF-α. We found that the gene expression of *Il1b*, *Il6* and *Tnfa*, three pro-inflammatory cytokines related to gzmA, was downregulated in the spinal cord or quadriceps from hemizygous mice. At protein level, reduced levels of IL1B and IL6 were found in quadriceps and the spinal cord, respectively, in both gzmA+/+ and gzmA+/− mice. Previous studies have analyzed the role of these pro-inflammatory cytokines in ALS pathology, showing associations between their absence and disease progression [35,36,37]. Particularly, increased levels of IL-1β, IL-6 and TNF-α in ALS patients compared to controls or patients with other neuropathies have been reported [12,38,39,40], suggesting that the modulation of these inflammatory mediators may ameliorate the course of the disease. In this line, a study reported that a deficiency of IL-1β extended the lifespan in SOD1G93A transgenic mice [41], which is in accordance with our results. Similarly, SOD1G93A mice lacking the receptor for advanced glycation end products (RAGE), a major component of the innate immune system involved in ALS pathogenesis, had slower disease progression and an extended lifespan, together with decreased expression of TNF-α and IL-1β in the spinal cord [42]. This could explain the longer survival rate observed in gzmA+/− mice, since gzmA can directly or indirectly activate the production of IL-1β, IL-6 and TNF-α [23,28]. In addition, the higher protein expression of IL1B and IL6 in gzmA−/− mice highlights that the presence of gzmA, even in lower levels, may be essential to regulate and maintain an optimal immune balance.

On the other hand, we found mRNA and protein levels of GSR reduced in the spinal cord and/or quadriceps of hemyzigous gzmA mice. GSR is an enzyme involved in oxidative stress, a key pathogenic mechanism dysregulated in ALS. In this sense, a wide variety of markers of free radical damage has been detected in blood, urine and cerebrospinal fluid (CSF) samples in ALS patients [43,44,45]. In ALS animal models with mutated hSOD1G93A, an increase in the levels of oxidation of different mRNA has been observed in the early stages of the disease, as well as a correlation between oxidation and a decrease in the expression of SOD1 [46]. Regarding gzmA, previous studies demonstrated that gzmA can activate the generation of reactive oxygen species (ROS) from mitochondria [47,48]. This could suggest that there could be reduced oxidative stress in gzma+/− mice, explained by the lower levels of GSR found in these mice. In contrast, the total absence of gzmA might not have a potential effect on oxidative stress, suggesting that gzmA may be partially needed in this mechanism. Developing therapies focused on decreasing oxidative stress in ALS have shown some positive outcomes, such as a treatment with curcumin that improved oxidative damage, showing a slight slowdown in disease progression [49], and AEOL 10150, a small molecule that catalyzes ROS [50,51]. In fact, the drug edaravone, recently approved for ALS treatment, is an antioxidant molecule able to alleviate the degeneration of both motor neurons and muscles in ALS patients [52,53].

Given the detrimental role of the dysregulated immune system in ALS, therapies focused on modulating immune cells and key inflammatory mediators, such as cytokines or T cells, would be of special interest. Over the last few years, numerous clinical trials have been developed based on this hypothesis [54,55]. One of the most promising drugs is masitinib, a tyrosine kinase inhibitor, which has shown positive effects in both animal models [56] and ALS patients [57]. However, to date, most of the therapies that have been successful in ALS animal models have hardly had any positive results when they have been carried out with patients. One of the reasons for this failure could be that most of the therapies have been focused on only one dysregulated pathological mechanism or even only one specific molecule. Given the multifactorial nature of ALS, where multiple molecular pathways are impaired, a potential therapeutic approach would be one targeting multiple molecular pathways. In this line, one study showed that the anti-aging and cognition-enhancing protein Klotho had neuroprotective, antioxidative, anti-inflammatory and promyelinating properties [58]. In this work, we have shown that modulation of gzmA can regulate some pro-inflammatory cytokines and one oxidative stress enzyme, suggesting that several pathways involved in the pathogenesis of ALS could be targeted when modulating gzmA. Supporting our results, a previous study reported that genetic variability of both inflammation and oxidative stress genes can affect the onset and the course of the disease in patients with ALS [59]. However, modulation of the immune system is challenging, as we have demonstrated in this study that a complete suppression of gzmA did not show the best results, but a partial reduction of gzmA was sufficient to modulate the gzmA-dependent pro-inflammatory cytokine response. The underlying cause may be based on ALS pathogenesis, which involves not only excessive inflammation, but also autoimmunity and inefficient immune responses [60]. In fact, gzmA seems to have a major and steady effect on regulating GSR. For this reason, therapies focused on the immune system and oxidative stress are promising in this disease, but the immunotherapeutic approaches should consider this dual role of the immune system in ALS pathogenesis.

In summary, we have demonstrated that a partial deficiency of gzmA may slightly ameliorate the progression of ALS disease, which could be explained by an effect on oxidative stress and a dual role of gzmA-mediated inflammation. Although these are only preliminary results and further research is needed to elucidate the precise role of gzmA in ALS pathogenesis, our results could shed light on the potential application of immunomodulation targeting gzmA as part of ALS therapy.

## 4. Materials and Methods

### 4.1. Animals

B6SJL-Tg SOD1G93A mice (stock number 002726, The Jackson Laboratory), inbred C57BL/6 mise deficient in gzmA (provided by Markus Simon from the Max-Planck-Institut für Immunbiologie, Freiburg) and their offspring were housed at the animal facilities in the Centro de Investigación Biomédica de Aragón. Their genotypes were analyzed as previously described for gzmA genotype [61] and following the The Jackson Laboratory protocol for *hSOD1G93A* gene.

All procedures were approved by the Ethics Committee for Animal Experiments at the University of Zaragoza (PI14/17). The care and use of animals were performed according to the Spanish Policy for Animal Protection RD53/2013, which meets the European Union Directive 2010/63 on the protection of animals used for experimental and other scientific purposes.

### 4.2. Crossings and Survival Study

**Figure 6:**
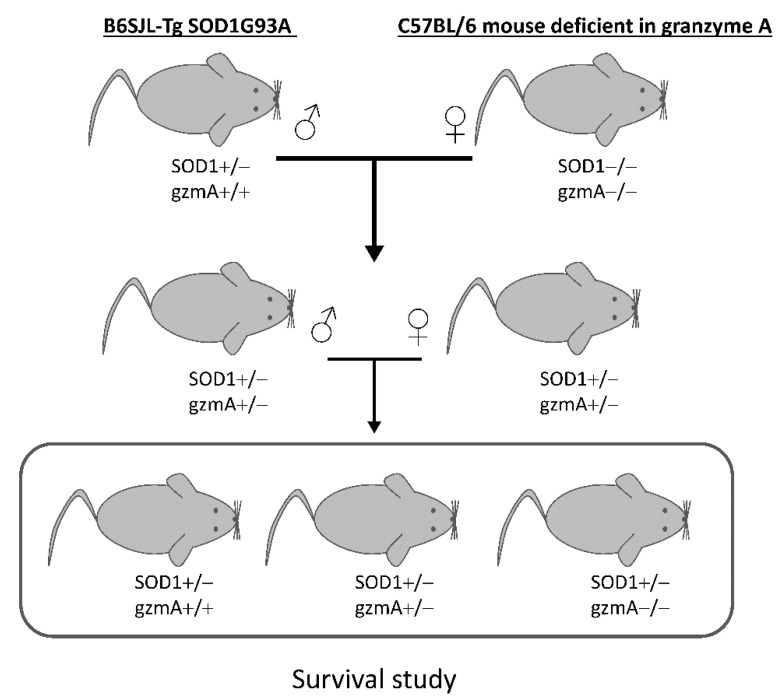
Scheme of crossings to generate homozygous and hemizygous gzmA mice. Hemizygous SOD1G93A males (SOD1+/−, gzmA+/+) were crossed with C57BL/6 females deficient in gzmA (SOD1−/−, gzmA−/−) to obtain hemizygous gzmA mice. Afterwards, we crossed hemizygous SOD1G93A and gzmA mice (SOD1+/−, gzmA+/−) to generate littermate mice with the three gzmA genotypes with the same genetic background.

Hemizygous SOD1G93A and gzmA mice (SOD1+/−, gzmA+/−) were obtained by crossing hemizygous SOD1G93A males (SOD1+/−, gzmA+/+) with C57BL/6 females deficient in gzmA (SOD1−/−, gzmA−/−). Next, we crossed hemizygous SOD1G93A and gzmA mice (SOD1+/− gzmA+/− males with SOD1−/− gzmA+/− females) to generate littermate mice with the three gzmA genotypes with the same genetic background (Figure 6). Survival analysis involved littermate sex-balanced hemizygous SOD1G93A mice (*n* = 34 gzmA−/−, *n* = 49 gzmA+/− and *n* = 19 gzmA+/+). Although the sample size for each group was different, we decided to include all the mice born in the survival study to obtain the greatest sample size. 

The humane endpoint for these mice used in the survival study was defined as the loss of the righting reflex, as shown by a failure to right after laying the mouse on its side for 30 s [34].

### 4.3. Enzyme-Linked ImmunoSorbent Assay (ELISA) for gzmA

Protein expression of gzmA was measured in serum samples from hemizygous SOD1G93A and gzmA mice. Blood samples were taken from 33 SOD1+/− mice, including gzmA+/+(n = 7), gzmA+/− (n = 14) and gzmA−/− (n = 12) mice at 90 days old, corresponding to the symptomatic stage of the disease, from cardiac puncture immediately after euthanasia. Blood was transferred to an Eppendorf tube and left undisturbed at room temperature for 30 min. Then, blood was centrifuged at 3000 rpm for 10 min at 4 °C, and supernatant serum was collected and immediately frozen in dry ice until being stored at −80 °C. 

A conventional indirect sandwich ELISA was performed to detect mouse gzmA, previously described [32]. Briefly, rat anti-mouse gzmA and rabbit anti-mouse gzmA antibodies were used to detect mouse gzmA. Specificity of these antibodies and ELISA was confirmed using cell lysates from wild-type (WT) and gzmA−/− animals. Purified rabbit IgG (2 µg/mL) was used as capture antibody, and rat polyclonal immune serum (diluted 1:1000 in PBS) was used as detection antibody. Highly absorbed goat anti-rat IgG (Sigma) labelled with peroxidase was used as secondary antibody diluted 1:10,000 in PBS, and TMB (Sigma) was used as chromogen. Absorbance was determined on the plate reader (Synergy™ HT, BioTek, Winooski, Vermont, USA) at 405 nm and 570 nm, and the values obtained at these wavelengths were subtracted (Abs 450-Abs 570).

### 4.4. RT-qPCR

Quadriceps and spinal cords were obtained from 30 littermate SOD1G93A mice (10 gmzA+/+, 10 gzmA+/− and 10 gzmA−/−) at the endpoint from the survival study. RNA was extracted using Direct-zol™ RNA MiniPrep kit (R2052, Zymo Research Corp., Irvine, CA, USA). Next, complementary DNA (cDNA) was synthesized from 1000 ng of total RNA using the qScript™ cDNA SuperMix kit (Quanta BioScience, Inc. Gaithersburg, MD, USA). Gene expression of *hSOD1G93A*, *Gzma*, *Il1b*, *Il6, Tnfa* and *Gsr* was quantified by qPCR using TaqMan^®^ probes and primers (Applied Biosystems Inc., Waltham, MA, USA) (Table 2). The data obtained were normalized with the housekeeping glyceraldehyde-3-phosphate dehydrogenase (*Gapdh*) (4352932E, Applied Biosystems Inc.). Mixes used for TaqMan^®^ probes and primers were TaqMan^®^ Fast Universal PCR Master Mix and Fast SYBR™ Green Master Mix (Applied Biosystems Inc., Waltham, MA, USA), respectively. Reactions were run in triplicate.

Thermocycling conditions were as follows: a holding stage of 20 s at 95 °C, then 40 cycles of 1 s at 95 °C, and 20 s at 60 °C for TaqMan^®^ assays; for SYBR™ assays, an initial step of 20 s at 95 °C, then 40 cycles of 3 s at 95 °C, and 30 s at 60 °C. The detected threshold cycle (Ct) values were normalized with *Gapdh* Ct values (ΔCt), and then ΔCt values were compared between samples in the statistical analysis. The ΔΔCT method was used to determine relative changes in transcriptional expression of *Gzma*, *Il1b*, *Il6*, *Tnfa* and *Gsr*. Graphs were performed using the fold change (2^−ΔΔCt^), as previously described [62]. 

### 4.5. Western Blot

Frozen spinal cord and quadricep muscle from 12 littermate SOD1G93A mice from the survival assay (4 gmzA+/+, 4 gzmA+/− and 4 gzmA−/−) were collected at the endpoint and ground into a powder using the Tissue Lyser LT (Qiagen, Hilden, Germany). Then, powdered tissues were resuspended in RIPA lysis buffer, together with protease inhibitors (SC-24948, Santa Cruz Biotechnology, Inc., Santa Cruz, CA, USA), according to manufacturer’s protocol. Total protein was quantified using the BCA method (Sigma-Aldrich, St. Louis, MO, USA). Next, a total of 30 μg of protein was loaded into a 10% SDS-PAGE gel to analyze the protein expression. After electrophoresis, proteins were transferred to a PVDF membrane (Amersham™, GE Healthcare Life Sciences, Marlborough, MA, USA) and subsequently blocked with a Tris-buffered saline solution containing 5% bovine serum albumin (BSA) and 0.1% Tween as supplement for 1 h at room temperature. Then, membranes were incubated overnight at 4 °C with the following primary antibodies: IL1B (sc-7884, Santa Cruz Biotechnology, Inc., Santa Cruz, CA, USA), IL6 (PK-AB815-61632M, Promocell, Heidelberg, Germany) and GSR (sc-32886, Santa Cruz Biotechnology, Inc., CA, USA). The housekeeping proteins selected to normalize the results were actin beta (ACTB) (PA1-183, Thermo Fisher Scientific Inc., Waltham, MA, USA) for spinal cord samples, and GAPDH (PK-AB718-3781, Promocell, Heidelberg, Germany) for muscle samples. Secondary antibodies used were goat anti-rabbit IgG (31466, Thermo Fisher Scientific Inc., Waltham, MA, USA) and m-IgG Fc BP-HRP (sc-525409, Santa Cruz Biotechnology, Inc., CA, USA). The dilution ranges used for each antibody are shown in Table 3. Finally, chemiluminescence detection was performed using Immobilon Crescendo Western HRP Substrate (Millipore, Billerica, MA, USA) in a Molecular Imager^®^ VersaDoc™ MP 4000 system. The computer-assisted analysis of the bands was performed with AlphaEase FC software (Bonsai Technologies Group, S.A., Madrid, Spain).

### 4.6. ELISA for TNF-α

Protein expression of TNFA was measured by ELISA using a TNF alpha Mouse Uncoated ELISA Kit (Catalog Number 88-7324, Invitrogen, Waltham, MA, USA) (Briefly, NUNC Maxisorp™ ELISA plate was coated with capture antibody against murine TNF alpha in Coating Buffer (100 μL/well) and incubated overnight at 4 °C. The following day, the plate was washed with wash buffer (0.05% Tween-20 in 1X PBS) and blocked with 200 μL of ELISA/ELISPOT Diluent (1X) for 1 h. Next, standards and samples were diluted in ELISA/ELISPOT Diluent (1X) and were incubated for 2 h at room temperature (100 μL/well). Later, plate was treated with biotin-conjugated anti-mouse TNF alpha antibody (Detection Antibody), exposed to Streptavidin-HRP (100 μL/well), and, finally, the reaction was read at 405 nm using a plate reader (Synergy™ HT, BioTek, Winooski, VT, USA). 

### 4.7. Statistical Analysis

The survival time of mice was evaluated using Kaplan–Meier analysis and Log-Rank test. After confirming the distribution of the data was normal according to Shapiro–Wilk test, comparisons of means of gene and protein expression were analyzed by unrelated t-test. Statistical analysis was performed using SPSS (version 20, IBM, Armonk, NY, USA), and graphs were made using GraphPad Prism Software (version 5, La Jolla, CA, USA). Significance levels were set at a *p*-value less than 0.05.

## Figures and Tables

**Table 1:** Descriptive statistics of the survival analysis for each group of transgenic mice.

Transgenic Mice Genotype	Mean (Days)	Median (Days)	SEM ^1^ (Days)	SD ^2^ (Days)	Minimum (Days)	Maximum (Days)
SOD1+/−, gzmA+/+	143.00	143.00	2.34	10.21	129.00	159.00
SOD1+/−, gzmA+/−	149.12	151.00	1.34	9.41	133.00	168.00
SOD1+/−, gzmA−/−	147.24	147.00	1.33	7.78	133.00	162.00

^1^ SEM: Standard error of the mean; ^2^ SD: Standard deviation.

**Table 2:** TaqMan^®^ probes and primer sequences used for qPCR analysis.

Genes	Type	Sequences/TaqMan^®^ Probe ID
*Gzma*	TaqMan^®^	Mm01304452_m1
*Il1b*	TaqMan^®^	Mm00434228_m1
*Il6*	TaqMan^®^	Mm00446190_m1
*Gsr*	TaqMan^®^	Mm00833903_m1
*Tnfa*	SYBR Green	Forward: 5′ TAT GGC CCA GAC CCT CAC A 3′
		Reverse: 5′ GGA GTA GAC AAG GTA CAA CCC ATC 3′
*hSOD1G93A*	TaqMan^®^	Hs00533490_m1
*Gapdh*	TaqMan^®^	4352932E

*Gzma*: granzyme A, *Il1b*: interleukin 1 beta, *Il6*: interleukin 6, *Gsr*: glutathione reductase, *Tnfa:* tumor necrosis factor alpha, *hSOD1G93A*: human mutant superoxide dismutase 1, *Gapdh*: glyceraldehyde-3-phosphate dehydrogenase.

**Table 3:** List of primary and secondary antibodies used in western blot.

Primary Antibody	Reference	Dilution	Secondary Antibody	Reference	Dilution	Tissue
ACTB	PA1-183, Thermo Fisher Scientific	1:1000	goat anti-Rabbit IgG	31466, Thermo Fisher Scientific	1:3000	Spinal cord
GAPDH	PK-AB718-3781, Promocell	1:1000	goat anti-Rabbit IgG	31466, Thermo Fisher Scientific	1:3000	Quadriceps
IL1B	sc-7884, Santa Cruz Biotechnology	1:250	m-IgG Fc BP-HRP	sc-525409, Santa Cruz Biotechnology	1:3000	Spinal cord, Quadriceps
IL6	PK-AB815-61632M, Promocell	1:1000	goat anti-Rabbit IgG	31466, Thermo Fisher Scientific	1:3000	Spinal cord, Quadriceps
GSR	sc-32886	1:500	goat anti-Rabbit IgG	31466, Thermo Fisher Scientific	1:3000	Spinal cord, Quadriceps

ACTB: actin Beta, GAPDH: glyceraldehyde-3-phosphate dehydrogenase, IL1B: interleukin 1 beta, IL6: interleukin 6), GSR: glutathione reductase.

## Data Availability

The data presented in this study are available on request from the corresponding author.
